# Accidental Hæmorrhage

**Published:** 1889-09

**Authors:** J. G. Swayne

**Affiliations:** Consulting Obstetric Physician to the Bristol General Hospital and Lecturer on Midwifery at the Bristol Medical School


### ACCIDENTAL HÆMORRHAGE.

Read at the Meeting of the Bath and Bristol Branch of the British Medical Association at Bath, May 30th, 1889.

BY

J. G. SWAYNE, M.D.,

*Consulting Obstetric Physician to the Bristol General Hospital and Lecturer on Midwifery at the Bristol Medical School.*

---

THE subject which I propose for our consideration this evening is a complication of labour that is generally called "Accidental Hæmorrhage." I have chosen it partly because it is a dangerous, although, happily a rare, complication; but chiefly because there are some points relating to it of which our knowledge is still somewhat uncertain and imperfect. I therefore hope that I may be able, from the results of my own experience, to add something, however small, to what we already know on the subject. Since I have been engaged in obstetric practice, I have met with eleven cases of Accidental Hæmorrhage. They are as follows:

1. In 1848, Mr. Crichton requested my father to see Mrs. P——, Regent Place, Clifton. My father being from home, I went instead, and found on my arrival that the patient had, during the eighth month of pregnancy, been suddenly attacked with hæmorrhage, occasionally severe and accompanied with labour-pains. She had lost a good deal of blood; her countenance was very pale and sunken and her pulse very weak and thready. The bleeding had been checked for a time by a large sponge, with which Mr. Crichton had plugged the vagina. On removing the sponge and examining, I found the os uteri dilated to the size of a half-crown; the membranes were unbroken, and I could pass my finger between them and the cervix uteri as far as I could reach, without feeling any placenta. In the anterior portion of the os, I could, however, feel a small foot with the toes pointed forwards; and as soon as the os was dilated to the size of a crown, could feel the heel and malleoli. I then ruptured the membranes, and, almost immediately with the escape of the waters, a large portion of apparently detached placenta came down into the posterior part of the os uteri. I was able, with some difficulty, to lay hold of the ankle and bring it down low enough to put a noose of tape round it, and then secure the other leg. As soon as the abdomen of the child appeared, I gave a second dose of ergot, the patient having taken ʒi. before I arrived. The child was then delivered. It was a small female child of seven months, which had apparantly been dead for some hours. The placenta was completely detached, and came away immediately after the child. The patient remained for some days in a state of great debility, but ultimately did well.

2. On March 18th, 1851, Mr. Ralph Bernard requested me to see Mrs. G——, living in York Buildings, a multipara whom he was attending. I was told that, two days previously, she was suddenly frightened by an accident which happened to one of her children, and ran quickly from one room into another; soon after this she was attacked with vomiting, followed by diarrhœa. On the next day hæmorrhage from the vagina came on, and continued at intervals, more or less profusely, until the evening, when Mr. Bernard saw her. He plugged the vagina and sent for me. On removing the tampon, I felt the os uteri soft and dilated to the size of a crown, the membranes being tense and protruding; a small moveable body could be felt in front, high up and behind the pubes, and behind and to the left of that, apparently the hand. When the os uteri was fully dilated and the membranes had descended to the perineum, we decided to interfere. I accordingly introduced my left hand into the vagina, for the purpose of rupturing the membranes, making out the presentation, and turning if necessary. Chloroform was given, with good effect, by Mr. Bernard, who had also previously given her gr. $\frac{1}{4}$ of acetate of morphia and gr. ij of acetate of lead, to check the bleeding. On rupturing the membranes, the head, which was small, soon descended into the pelvic cavity, the hand was pushed above it, and labour soon terminated. The child was a seven months' child and was stillborn, having been apparently dead for two or three days. The mother did well.

3. In 1854, I attended Mrs. S——, a multipara, living at Cotham. The labour came on about two months before the time, being brought on, as she supposed, by the straining occasioned by a violent cough from which she was suffering, and which afterwards proved to be whooping cough. The commencement of labour was indicated by a slight amount of hæmorrhage, unaccompanied with pains; and this continued until the middle of the next day; the labour making little or no progress, and the os uteri being barely large enough to admit the tip of the finger. I gave her an opiate in hopes of arresting the labour. It did not, however, succeed; for in about three hours more hæmorrhage ensued, several clots were expelled, and the pains became sharp and frequent. In about an hour and a half more, a stillborn seven-months'

fœtus was expelled. The placenta followed almost immediately. On the next day, after several severe after-pains, a firm clot, as large as two fists, was expelled. The patient was at first rather weak from loss of blood, but ultimately did well.

4. On November 3rd, 1861 (about a month before her expected time), I was sent for to see Mrs. L——, a primipara, living on Cotham Hill, who was attacked with severe hæmorrhage after taking a walk. This was checked by strict rest and by giving an opiate, followed by small doses of turpentine. The hæmorrhage returned on November 22nd, and could not be checked by the same means. Labour commenced and continued, accompanied with bleeding between the pains. Opiates were first given; after that a dose of ergot, and a binder was applied, as the bleeding was still rather profuse, and the os uteri was dilated to the size of a crown, the membranes covering the head presenting, I ruptured them with my finger. This immediately checked the bleeding, and the labour soon terminated in the birth of a stillborn male child. There was much debility after the confinement, and it was necessary to give tonics, quinine, iron, &c.; but after a time the patient did well.

5. On June 19th, 1869, Mr. Burleigh asked me to see with him, Mrs. S——, St. Matthew's Road, Kingsdown. She was the mother of five children, and in her last confinement nearly died from *post partum* hæmorrhage. She was, moreover, a person of intemperate habits. Mr. Burleigh told me that she was attacked with violent bleeding early in the labour, when the os uteri was scarcely open. He had controlled this in some degree, by applying cold, by plugging, and by giving ergot; but yet a great oozing of blood was going on. I arrived at 11 p.m.; she was then very weak from loss of blood. P. 140. I withdrew the plug, and on examining I found the os to be about the size of a florin, and the bag of the waters presenting. As there was then no hæmorrhage we waited about an hour; but as there was little or no pain and the case was getting urgent, I examined again, and found the os dilated to the size of a half-crown, but still rather rigid. I could feel a foot through the membranes. I then ruptured the membranes and brought down the presenting foot, and afterwards the other, and the rest of the child soon followed. It was a male, stillborn, and apparently about a month before the full time. The placenta was expelled immediately after the birth of the child, together with several large dark clots. In spite of all our attempts to rally the patient she gradually sank, and died about an hour after delivery.

6. On October 4th, 1873, Mr. Talbot asked me to see a poor woman living in Frog Lane, who was being attended by a midwife. She had had one child previously. I saw her at eight a.m. She was much blanched and the pulse was very feeble. The hæmorrhage had been profuse. On examining, I found the os uteri about the size of a shilling and rather rigid. I could feel the membranes and the head presenting; but no placenta. There was no hæmorrhage with the pains, which were regular, but not strong. The midwife had ruptured the membranes during an examination. I gave ʒi. of liquid extract of ergot, and ʒiij. of turpentine, and introduced a Barnes' bag (No. 1.) to dilate the os uteri. We then left for two hours. On our return the midwife told us that vomiting had come on, which was followed by strong pains, which expelled the bag and the child about 11.30 a.m. The child was stillborn, and had apparently been dead for some days. The placenta must have been attached to the fundus uteri, for the opening in the membranes which gave exit to the child, was just opposite to it. The woman made a good recovery.

7. Mr. Davies requested me to see with him Mrs. U——, living in West Street. She had had three children previously, and was about eight months pregnant. On the preceding day (March 17th, 1875) she experienced severe pain in the back and thighs whilst walking in town, and on her return home was seized with bleeding from the vagina and faintness. I found on examination the os uteri dilated to the size of a shilling, and could feel the head through the anterior wall of the uterus. The membranes were tense and protruding. As there was at present no hæmorrhage, I advised delay for a short time before rupturing the membranes. This was done by Mr. Davies, after a short rest, and in about an hour the child was born; it appeared to have been dead for some hours. There was no more hæmorrhage, and the mother did well.

8. On December 14th, 1875, Mr. Ellis called me in to Mrs. S——, a multipara, living at Melbourne Villa, Cotham Road; she had then been just confined. I had seen her with him some days before, because there had been been a considerable flow of blood from the vagina, apparently proceeding from some detachment of the placenta; but as there was then scarcely any dilatation of the os tincæ, and the bleeding had quite ceased, we did nothing. Labour came on naturally in a few days, and she was delivered of a large stillborn male child. Mr. Ellis said she was very faint at the time of delivery, and as the placenta did not come away he sent for me. When I arrived I found her still rather faint, and requiring stimuli frequently. The placenta had been retained an hour and a half. I therefore introduced my hand and peeled it off, with some difficulty, for it was extremely adherent. This was accompanied with some further loss of blood, and she lay for two hours in a very exhausted and pulseless condition. We compressed the uterus, gave a full dose of ergot, and frequent stimuli until she rallied, and after three hours appeared to be doing well. But, about four days after this, symptoms of septicæmia began to show themselves; and as I was attending other cases, Dr. Martyn attended in consultation, instead of me. She had a long tedious illness, in the course of which she had phlegmasia dolens of both legs, pleurisy, and extensive bed-sores, and finally sank, and died on January 17th, 34 days after delivery.

9. On March 28th, 1886, I attended Mrs. S——, Northcote Road, in her third confinement. Labour began about 1 a.m. with a discharge of bright red clots, followed by dull aching pains in the bowels and joints; but not regular labour-pains. This was afterwards followed by some more discharge of fluid blood. I examined and found the os uteri dilated to the size of a half-crown. The membranes covering the head were presenting. I at once ruptured them with my finger, and gave ʒi. of liquid extract of ergot. This checked the hæmorrhage, regular pains came on, and the child was born at 7.30 a.m. It was a female child, stillborn; all efforts to restore it were useless. After this the placenta was expelled, together with some fluid blood and clots. The patient did well.

10. About 1 a.m., April 3rd, 1886, Dr. Elliott summoned me to a case he was attending: Mrs. E——, a primipara living in Anglesea Place. When I arrived

I found her pulseless and in a state of collapse. There had been copious uterine hæmorrhage, and the bed-linen was saturated with blood. Dr. Elliott told me that on the previous day he had examined her urine, and found, that when heated, there was an albuminous deposit, which formed two-thirds of the whole amount. He said that labour had begun with copious hæmorrhage; but yet without proper labour-pains, and that he had ruptured the membranes. There was but little fresh hæmorrhage after my arrival. The os uteri was dilated to the size of a half-crown. Dr. Elliott had tried to introduce the forceps, to hasten delivery; but the os uteri was not large enough to admit both blades. As the collapse was very great, and as she had taken plenty of brandy, I injected ʒi. of sulphuric ether hypodermically, whilst Dr. Elliott applied hot flannels to the region of the heart. However, she did not rally, but sank, and died at 2 p.m. Whilst she was *in articulo mortis*, Dr. Elliott turned and delivered her of a stillborn female infant, and also brought away the placenta.

11. On February 14th, 1889, Dr. Colman sent for me to see Mrs. W——, Waverley Road, a primipara, aged 19, whom he was attending. On arriving he told me that labour commenced with a gush of blood, which suddenly came from her whilst walking in her bedroom. Regular pains then came on, and the labour was tolerably expeditious. He noticed, however, a waxy pallor of the countenance during labour. Soon after this a copious bleeding took place, when the placenta was expelled. When I arrived she had rallied and was rather better. The uterus was well contracted, and there was no more hæmorrhage. The pulse was very feeble, the countenance very pale, and there was severe nausea. I gave her a dose of ergot, and also sal-volatile and ether, and tightened the binder. The child, which was apparently little short of the full time, was born alive and did well, and the mother also made a fair recovery. Dr. Colman told me that the husband attributed the hæmorrhage to sexual intercourse, which had taken place a short time previously.

*Causes.*—In England, the term "Accidental Hæmorrhage" is generally applied to that form of *ante partum* hæmorrhage of which I have just given cases: but on the Continent, and especially in France, this term is not in general use; chiefly, I think, because the French differ somewhat from us as to the cause of this complication. In England the hæmorrhage is considered to be usually due to an accidental detachment of a portion of the placenta, and to be occasioned by much the same causes as would produce abortion during the earlier months of pregnancy: such as shocks, either mental or bodily; falls, or blows on the abdomen; excessive muscular exertion, either voluntary or reflex, such as coughing, sneezing, or vomiting. These are, I think, the most frequent exciting causes; but there are others, such as irregular contractions of the uterine muscular fibres, which disturb and break the relations of surface between the uterus and placenta; and if these contractions are sudden or excessive, the placenta may be partially loosened. And, thirdly, uterine congestion: any sudden determination of blood to the uterus and placenta may cause detachment of the latter. In France more importance is attributed to this last exciting cause than in England, and most French authors consider that the hæmorrhage precedes and is the cause, rather than the consequence, of the placental detachment. Velpeau compared it to epistaxis resulting

### CASES OF ACCIDEN[illegible]

| No. | Date. | Name, &c. | No. of Pregnancy | Original Attendant. | Presentation. | Causes and Complications. |
|---|---|---|---|---|---|---|
| 1 | 1848. | Mrs. P., Regent place | Multipara | Mr. Crichton | Feet | Uncertain |
| 2 | March 18th, 1851. | Mrs. G., York buildings | Multipara | Mr. R. M. Bernard | Head and Hand | Apparently caused by a fright |
| 3 | 1854. | Mrs. S., Cotham hill | Multipara | Dr. Swayne | Vertex | Caused by whooping cough |
| 4 | Nov. 3rd, 1861. | Mrs. L., Cotham hill | 1st | Dr. Swayne | Vertex | Caused by too long walk |
| 5 | June 19th, 1869. | Mrs. S., St. Matthew's road | 6th | Mr. Burleigh | Feet | Intemperance. |
| 6 | Oct. 4th, 1873. | Mrs. — Frog lane | 2nd | Midwife and Mr. Talbot | Vertex | Uncertain |
| 7 | March 18th, 1875. | Mrs. U., West street | 4th | Mr. Davies | Vertex | Caused by too long walk |
| 8 | Dec. 14th, 1875. | Mrs. S., Cotham road | Multipara | Mr. Ellis | Vertex | Adherent Placenta, which had to be removed |
| 9 | March 28th, 1886. | Mrs. S,, 4 Northcote road | 3rd | Dr. Swayne | Vertex | Uncertain |
| 10 | April 3rd, 1886. | Mrs. E., Anglesea place | 1st | Dr. Elliott | Vertex | Albuminuria previous to labour |
| 11 | Feb. 14th, 1889. | Mrs. W., Waverley road | 1st æt. 19 | Dr. Colman | Vertex | Caused by sexual intercourse |

### HÆMORRHAGE.

| Treatment. | Result to Mother. | Result to Child. | REMARKS. |
|---|---|---|---|
| Plugging. Ergot. [illegible]pture of Membranes. | Recovered | Dead | Seven months' child. Apparently dead for some hours. |
| Opiates and Astringents. [illegible]pture of Membranes. Hand pushed up. | Recovered | Dead | Seven months' child. Apparently dead for some days. |
| Opiate at first. [illegible]xpelled by natural efforts. | Recovered | Dead | Seven months' child. |
| Ergot and Turpentine. Binder. [illegible]pture of Membranes. | Recovered | Dead | Large child. Nearly full time. |
| Plugging. Ergot. [illegible]upture of Membranes. | Died an hour after delivery. | Dead | Eight months' child. |
| Turpentine. Barnes' bags. Rupture of Membranes. | Recovered | Dead | Child apparently dead for some days. |
| Rupture of Membranes. | Recovered | Dead | Apparently dead for some hours. |
| Delivered naturally. | Died after 34 days | Dead | Septicæmia came on fourth day, December 17th, and she died January 17th, 1876. |
| Ergot and Rupture of Membranes. | Recovered | Dead | Child had apparently been dead some hours. |
| Injections of Ether. Rupture of Membranes. Turning. | Died immediately | Dead | |
| Delivered naturally. [illegible] and stimuli given afterwards. | Recovered | Living | |

from excessive congestion of the mucous membrane of the nose; and Cazeaux asks: "Is the separation of the placenta the primitive phenomenon, and has it caused a vascular rupture? or has this rupture taken the precedence, and has the effusion of blood between the after-birth and the uterus, resulting therefrom, produced the separation of the placenta? The latter opinion appears to me the more probable." *

The cases I have just brought forward tend, I think, to prove that the English view is probably the more correct one. In five, the hæmorrhage immediately followed a very evident exciting cause. For instance, in two cases (4 and 7) out of the eleven it followed a long walk. In one case (3) it was caused by a paroxysm of whooping-cough, in another (2) by sudden exertion caused by fright, in another (11) by sexual intercourse—a most likely cause of excessive uterine congestion and irregular uterine contractions. In most of my other cases the causes are predisposing rather than exciting. Before alluding to these, I shall quote *verbatim* from Barnes's *Obstetric Medicine and Surgery*† some excellent remarks upon this head: "This premature separation of the placenta rarely occurs in the young and robust, differing in this respect from the case of placenta prævia. It is most common in women past thirty-five years of age, who have borne many children, whose constitutions are worn by sickness and poverty, perhaps by intemperance, whose tissues are thereby badly nourished, wanting in tone, tending to atrophy or degeneration; in short, in the same class of persons who are most liable to rupture of the uterus. In one case we found fatty degeneration of the heart. Certain diseases dispose to hæmorrhage, and notably to

* Cazeaux's *Midwifery*, p. 753. † Vol. II., p. 238.

this form. Variola, albuminuria (Blot), leucocythemia (Paterson), acute atrophy of the liver, are amongst the most potent. A strong predisposing cause may exist in *a morbid condition of the placenta*, such as fatty degeneration, fibrinous masses, or atrophy, by which the uniformity of its structure is impaired. A diseased placenta, especially one of unequal consistency, containing solid lumps, will not follow and adapt itself to the varying movements of the uterus, and the changing superficial area of the the placental site, so easily as a healthy placenta does."

In proof of the correctness of this description of Dr. Barnes, I will refer to the three worst, in fact, to the three fatal, cases in my series. In all of these a morbid condition of the blood was the predisposing cause. In case 5, this was due to intemperate habits; in case 8, there was fatty degeneration and partial adhesion of the placenta; and in case 10, well-marked albuminuria.

There are still three cases (Nos. 1, 6, and 9) in my series, in which no evident exciting or predisposing cause was observed. Probably, local congestion may have preceded and occasioned the bleeding, or possibly the lowest edge of the placenta may have been attached just within the lowest or dangerous zone of the uterus, as described by Barnes, but so high as to be out of reach of the finger. Such a case would really be one of partial placenta prævia. Still, however, notwithstanding these anomalous cases, there is, I think, abundant evidence to justify the use of the term "Accidental Hæmorrhage."

*Frequency.*—There is some discrepancy of evidence as to the frequency of *Accidental Hæmorrhage.* All statistics show that it is a rare complication of labour, just as *Unavoidable Hæmorrhage* is; but authorities differ as to the relative frequency of accidental and unavoidable hæmorrhage. On the whole, the most reliable statistics are those which are chiefly obtained from large lying-in hospitals, and not from private practice. Dr. Churchill gives a list of this kind, amounting in all to 92,424 cases. In these there were 251 cases of accidental, and 124 of unavoidable, hæmorrhage; or rather more than twice as many of the first kind. In my own practice, however, I have met with 11 cases of the former, and 23 of the latter, a result that would at first sight lead to just the opposite conclusion. But the statistics of private practice are very apt to lead to false conclusions, unless one carefully eliminates all the consultation-cases. If I treat my own cases in this way, I find that in 3 of the 11 of accidental hæmorrhage I was the original attendant, and in only one of the 23 of unavoidable hæmorrhage; thus showing a still greater preponderance of the former. The reason why there are so many more consultations in cases of placenta prævia, than in accidental hæmorrhage is, that the treatment of the latter is comparatively free from doubt or difficulty; whereas that of the former tries to the utmost the nerve, judgment, and skill of the practitioner first in attendance, and he therefore, very wisely, requests a consultation. After carefully excluding all consultation-cases, I find that I have met with 3 cases of accidental hæmorrhage in a total of 1,539, or exactly 1 in 513 cases. This is a rather greater frequency than is met with in the Guy's Hospital Lying-in Charity, where, according to Dr. Galabin, it is about 1 in 760 cases.

In by far the greater number of cases the nature of the accident is soon apparent by an external escape of blood, before any serious symptoms of collapse are manifest. A portion of the placenta becomes detached, and blood becomes effused between the membranes and the uterus.

This is usually accompanied with dull pains, like those of colic in the region of the fundus uteri; and also some irregularity in the shape of the fundus. But the effused blood soon finds its way to the os uteri, and escapes externally. The nature of the case is then apparent, especially if the os uteri be more or less dilated. The history of the case, the inability to feel any portion of the placenta within the os; if labour-pains are present, the fact that they check rather than increase the bleeding —all these symptoms will serve to point out the nature of the case, and to render the diagnosis of accidental from unavoidable hæmorrhage comparatively easy, and the prognosis comparatively favourable. Most of my own cases were of this kind. But in other and fortunately much rarer instances, there are little or no signs of labour, the os uteri remains nearly closed; it is a long time before blood appears externally, and before it does, severe constitutional symptoms of hæmorrhage have set in. In these the diagnosis is more difficult, and the prognosis less favourable. Some of the cases I have given partook more of this character, and were attended with symptoms more or less critical. But the worst cases of all are those of what is called *concealed hæmorrhage*. In these there is no external show of blood; but there are dull and obscure pains in the belly like those of colic, combined with tension and swelling of the fundus uteri, and succeeded by pallor of countenance, failure of pulse, sighing respiration, vomiting, syncope, and extreme collapse, so that the patient may actually die undelivered, and the true nature of the case be unsuspected by an inattentive or inexperienced practitioner. It may be mistaken for a severe case of syncope, arising from that common excuse for ignorance, a "weak heart;" and, in consequence of the popular prejudice that exists against any *post mortem* examination of a woman who dies undelivered, the truth may never be discovered. In these cases the detachment usually begins in the centre of the placenta, and proceeds towards the circumference, until a large amount of blood accumulates between the placenta and membranes and the uterine wall, distending the latter almost to rupture, and inducing an amount of shock which increases still further the previous collapse. In this way the placenta may be more completely separated from the uterus than it is in placenta prævia, without giving the same obvious signs of danger. No proper labour-pains come on, because the shock caused by the blood-pressure renders irregular, perverts and even paralyses the normal uterine contractions. This form of accidental hæmorrhage is nearly always the result of great constitutional debility, and is happily exceedingly rare. I have never met with a well-marked case. Dr. Galabin records only one out of a total of 23,591 deliveries in the Guy's Hospital Lying-in Charity. Dr. Goodell of Philadelphia, however, succeeded in obtaining from every available source a record of no less than 106 of these critical cases, which he published in the *American Journal of Obstetrics* for 1869. Of these 106 cases, 54 or rather more than half the mothers perished; and of 107 infants, 6 only were known to have been saved. Thus the mortality to the mothers was nearly 51 per cent., and to the children 94 per cent.; a mortality to both which far exceeds that from placenta prævia.

In the ordinary run of cases of accidental hæmorrhage the prognosis is not nearly so grave. According to Dr. Churchill's statistics, the maternal mortality is nearly one in four; in Dr. Galabin's, nearly 1 mother in 6 died of hæmorrhage. This is much less than the mortality in cases of placenta prævia. In my own 11 cases, there were 3 maternal deaths; or nearly 1 in $3\frac{1}{2}$, which, according to Dr. Churchill, is the usual maternal mortality in placenta prævia, and is higher than that which is generally observed in accidental hæmorrhage. But then it should be noticed that one of the deaths in my list occurred 34 days after delivery, and was occasioned not by hæmorrhage, but by septicæmia; whereas another was in a great measure due to albuminuria.

The statistics as to the infant mortality in accidental hæmorrhage are, I find, very incomplete and not very reliable. I cannot get any information from Dr. Churchill's on this point. This kind of hæmorrhage is generally considered to be quite as dangerous to the child as that proceeding from placenta prævia. My own experience proves it to be still greater. In my 11 cases only one child lived. The rest died; nearly all being born dead, and beyond all chance of resuscitation. Most of them, judging from appearances, looked as if they had been dead for some hours. Dr. Barnes remarks that, "the prospect of the child's survival depends greatly on the extent of the detachment of the placenta, and the time that elapses before its extrication from the womb." * Now, as regards the first of these conditions, I believe that the placenta at birth is often more detached in accidental than in unavoidable hæmorrhage. In several of my cases of the former, the same pain that expelled the child, brought away also the placenta and several clots; the placenta appearing as if it had been quite detached for some time, and lying loose in the cavity of the womb. Again, the time that elapses before the placenta

* *Obstetric Medicine and Surgery*, Vol. II., p. 241.

is extracted from the womb, is generally much greater in accidental than in unavoidable hæmorrhage. In the former, especially if the result of a shock or fall, the hæmorrhage often begins before the os uteri is at all dilated; but in the latter there is nearly always some dilatation before the bleeding becomes dangerous; and when it is so, the practitioner delivers as quickly as he can with safety to the mother; and this very prompt delivery also gives the child a better chance of life. Whereas in accidental hæmorrhage, the detachment of the placenta takes place very early, or even precedes the labour, and is not usually necessary or even advisable, if we consider the safety of the mother, to deliver so promptly, or even to interfere at all after the membranes are ruptured. But this very delay, although perhaps to the advantage of the mother, is a sort of additional danger to the child; for when its connection with the mother is almost, perhaps quite, severed, the sooner it is delivered the better chance it has of living. The reasons I have given will, I think, account for the fact that in my 11 cases of accidental hæmorrhage only 1 child was saved; whereas, in the 23 I have recorded of placenta prævia, as many as 5 were born alive. Moreover, as far as I have seen, the child is more apt to be born prematurely in accidental than in unavoidable hæmorrhage. I am therefore led, as far as my own experience goes, to regard accidental hæmorrhage as fraught with greater danger to the child than perhaps any other complication of labour.

With regard to the *presentation*. In the 11 cases I recorded it was abnormal in three; of this the feet presented in two, and the head and hand in one. In 23 cases of placenta prævia which I have recorded, there were six abnormal presentations: so that in both rather more than a quarter were abnormal presentations. Although the tendency to this departure from the ordinary course of nature appears to be equal in both of these complications, yet it is greater, I think, than can fairly be attributed to premature delivery only.

With respect to *treatment*, I find that, in 3 cases out of the 11, delivery took place naturally. The labour was quick, and the symptoms were not so urgent as to render active interference necessary. In 3, plugging of the vagina was resorted to on the appearance of hæmorrhage. In each case this had been done, before my arrival, by the practitioner previously in attendance. I have myself never had recourse to the vagina plug, in such circumstances; for I distrust it very much in cases of accidental hæmorrhage. It merely hides the danger; it does not arrest the flow of blood, but merely prevents its external manifestation; whilst all the time it is in use, blood may be accumulating within the uterus to a fatal extent. In placenta prævia the vagina plug is comparatively safe; for it presses on that part of the uterine neck from which the placenta is already detached, whilst, at the same time, the adherent portion of the placenta above this point prevents the blood from finding its way back into the uterine cavity. The only kind of plug which I consider admissible in cases of accidental hæmorrhage is one which is used to dilate a small and rigid os, so as to prepare the way for further operations; such a plug, for instance, as a sponge-tent, or, what is much better, one of Dr. Barnes's hydrostatic bags; but whilst these are in use the patient should be carefully watched. In only one of my cases was it necessary to use one. Medicines, as a general rule, are not of much service in accidental hæmorrhage, except when given early, before the symptoms are urgent, and when we wish to gain time. For this purpose opiates, and astringents, such as the acid infusion of roses, acetate of lead, and turpentine, may be used with good effect, when combined with the most perfect rest in the recumbent posture, and the application of cold, either by wet pads or, what is better, an India-rubber bag containing ice placed over the fundus uteri. In five of my cases such measures were employed; but I cannot say that their effect appeared very decided. The remarks I am making, however, do not apply to another medicine that may be used with much better effect later on, and that is the ergot of rye. The fact is, that in bad cases of this kind, we must trust to our hands and not rely upon drugs. The remedy, above all others, to be adopted is one which we owe to Puzos, a French accoucheur who lived in the last century; and that remedy is *rupture of the membranes*. As Dr. Barnes remarks: "The first thing to do in all the cases is to rupture the membranes. This, by letting off the liquor amnii, takes off the strain on the uterine fibre, allows the walls to resume their natural condition, and provokes labour." Should there be great prostration, it will be well, before resorting to this or any kind of manual interference, to endeavour to rally the patient (as in case 10) by a hypodermic injection of ʒi. of sulphuric ether, which may be repeated every half hour if necessary. Should the prostration be accompanied with a great want of uterine action, we should endeavour to remedy this by applying an abdominal binder, and gradually tightening it over the fundus uteri; at the same time that we give two or three doses of ergot either by the mouth or by subcutaneous injection. If the os uteri is so small and rigid as not to admit of further measures, it should first be dilated by Barnes's bags until it is large enough either to admit the hand or forceps without difficulty. Forced delivery in these cases is dangerous, and always, if possible, to be avoided. It inflicts a shock on the system which is likely to increase the previous prostration, and it may cause fatal laceration of the uterine neck; but, if after sufficient gradual dilatation of the os uteri, effectual labour-pains do not set in, delivery must be accomplished either by turning or the forceps, according to the judgment of the accoucheur as to which operation can be performed with least difficulty, and with the smallest amount of shock. After the birth of the child, the greatest care should be taken to ensure proper uterine contraction, and guard against the occurrence of *post partum* hæmorrhage, a very small amount of which may prove fatal to a patient who has already lost a large quantity of blood. In conclusion, I may say that in the majority of cases of accidental hæmorrhage, it will be sufficient to control the bleeding by rupturing the membranes; after this the rest of labour may be left to nature: but in the minority of cases, which are more severe, and accompanied with greater prostration and greater want of uterine power, it will be necessary to terminate the labour artificially. In selecting the best mode of accomplishing this, the safety of the mother is the first, indeed I may say the only, consideration; that of the child is already compromised by the hæmorrhage to such an extent, and the chance of saving its life is so extremely small, that the accoucheur would be justified in resorting even to craniotomy (as recommended by Dr. Robert Lee in such cases), if he thought that he could thereby deliver the mother with less shock, with greater ease, and therefore with greater safety.

